# Prolonged Subcutaneous Administration of Oxytocin Accelerates Angiotensin II-Induced Hypertension and Renal Damage in Male Rats

**DOI:** 10.1371/journal.pone.0138048

**Published:** 2015-09-22

**Authors:** James Phie, Nagaraja Haleagrahara, Patricia Newton, Constantin Constantinoiu, Zoltan Sarnyai, Lisa Chilton, Robert Kinobe

**Affiliations:** 1 College of Public Health, Medical & Veterinary Sciences, James Cook University, Townsville, Queensland 4811 Australia; 2 Centre for Biodiscovery & Molecular Development of Therapeutics, Australian Institute of Tropical Health and Medicine, James Cook University, Townsville Queensland 4811, Australia; University Medical Center Utrecht, NETHERLANDS

## Abstract

Oxytocin and its receptor are synthesised in the heart and blood vessels but effects of chronic activation of this peripheral oxytocinergic system on cardiovascular function are not known. In acute studies, systemic administration of low dose oxytocin exerted a protective, preconditioning effect in experimental models of myocardial ischemia and infarction. In this study, we investigated the effects of chronic administration of low dose oxytocin following angiotensin II-induced hypertension, cardiac hypertrophy and renal damage. Angiotensin II (40 μg/Kg/h) only, oxytocin only (20 or 100 ng/Kg/h), or angiotensin II combined with oxytocin (20 or 100 ng/Kg/h) were infused subcutaneously in adult male Sprague-Dawley rats for 28 days. At day 7, oxytocin or angiotensin-II only did not change hemodynamic parameters, but animals that received a combination of oxytocin and angiotensin-II had significantly elevated systolic, diastolic and mean arterial pressure compared to controls (P < 0.01). Hemodynamic changes were accompanied by significant left ventricular cardiac hypertrophy and renal damage at day 28 in animals treated with angiotensin II (P < 0.05) or both oxytocin and angiotensin II, compared to controls (P < 0.01). Prolonged oxytocin administration did not affect plasma concentrations of renin and atrial natriuretic peptide, but was associated with the activation of calcium-dependent protein phosphatase calcineurin, a canonical signalling mechanism in pressure overload-induced cardiovascular disease. These data demonstrate that oxytocin accelerated angiotensin-II induced hypertension and end-organ renal damage, suggesting caution should be exercised in the chronic use of oxytocin in individuals with hypertension.

## Introduction

Recent advances indicate that oxytocin (OT) is potentially a useful therapeutic agent for many neuropsychiatric conditions including autism, social anxiety, postnatal depression, obsessive-compulsive problems and schizophrenia [1, 2]. However, the vast majority of studies on the putative use of OT as a therapeutic agent have been based on a wide range of single or short-term OT doses in normal individuals and thus failed to determine how acute and chronic OT administration differ, and how the therapeutic effects of OT in normal individuals may translate to patients with comorbidities of brain disorders and other systemic disease conditions [3]. The oxytocinergic system influences physiological functions in many organ systems outside the central nervous system. OT is synthesised in the heart and the OT receptor similar to that found in the uterus and brain was identified in mammalian cardiac myocytes, cardiac fibroblasts and blood vessels [4, 5]. The first cardiac specific role of OT was demonstrated by its ability to trigger the release of atrial natriuretic peptide (ANP) from mammalian hearts [6], causing natriuresis and a decrease in blood pressure [7]. Similarly, OT is associated with a direct, centrally mediated regulation of autonomic outflow to the heart leading to negative inotropic and chronotropic effects [8, 9]. In experimental models of ischemic cardiac injury, single sub-pressor doses (25–125 ng/Kg) of OT significantly reduced myocardial infarct size, improving left ventricular function by activating cytoprotective intracellular mechanisms including the stimulation of endothelial nitric oxide synthase-guanylate cyclase, ANP-cyclic guanosine monophosphate, phosphoinositide 3-kinase and protein kinase B pathways likely via the OT receptor [10–12]. In the heart, these intracellular signalling mechanisms subserve many beneficial functions including vasodilation, anti-apoptotic and anti-fibrotic effects [10, 13]. It has also been suggested that the protective effects of OT in myocardial ischemic injury are linked to ischemic preconditioning [10, 11]. Acute stimulation of the cardiac OT receptor caused the up-regulation of protein kinase C, which triggers the activation of mitochondrial ATP-dependent potassium channels (mitoK_ATP_) [10, 13]. This elicits cardiac protective effects through increased potassium ion uptake into the mitochondria, leading to reduced mitochondrial steady-state matrix volume, respiratory uncoupling and matrix alkalization [14]. Opening of mitoK_ATP_ channels also causes sustained production of reactive oxygen species during the ischemic period but reduces it following reperfusion thus preventing irreversible opening of the mitochondrial permeability transition pore [15].

In marked contrast however, the acute short-term and specifically targeted benefits of OT could be negated leading to unpredictable outcomes if the use of this neuropeptide is prolonged in chronic cardiovascular conditions such as hypertension and pressure overload-induced cardiac and renal damage. For instance, in ischemic cardiac injury, a short-term targeted administration of OT increased the expression of transforming growth factor-beta1 in the infarct and was associated with improved cardiac function and decreased mortality [11, 16, 17]. However, chronically increased transforming growth factor-beta 1 expression causes pathological cardiac remodelling characterised by hypertrophy, apoptosis and fibrosis [18]. This demonstrates that possible chronic effects of OT on cardiovascular function cannot be inferred from studies with acute dosing regimens. Furthermore, activation of the OT receptor is known to increase intracellular calcium concentration mediated by the stimulation of phospholipase C as seen in the myometrium [19, 20]. In the heart, accumulation of intracellular calcium and the subsequent activation of calcium dependent calcineurin activity, and its downstream effector, the nuclear factor of activated T-cells (NFAT) is a hallmark and a critical intracellular signalling convergence point for many pathological conditions, including chronic pressure overload-induced cardiac hypertrophy and failure, and ischemic injury [21, 22]. Mammalian myometrial cells and cardiac myocytes share an identical OT receptor [23] but OT treatment appears to ameliorate endothelin-1 and Angiotensin II (AngII) mediated NFAT activation, hypertrophy and apoptosis in neonatal rat cardiac myocytes [24]. While this *in vitro* study based on neonatal cardiac myocytes indicates that OT may attenuate the effects of pressure-overload induced cardiac hypertrophy and pathology, effects of prolonged OT administration on cardiovascular health in general, and chronic pressure overload-induced cardiac hypertrophy and end-organ damage *in vivo* have not been investigated. We hypothesised that in the presence of a chronic hypertensive state, prolonged administration of OT may be pathological. Our objective was to examine effects and putative pathophysiological mechanisms of prolonged administration of low dose OT in a rodent model of AngII-induced hypertension, cardiac hypertrophy and renal damage.

## Materials and Methods

### Chemicals and Reagents

OT was obtained from Bachem (Bubendorf, Switzerland), AngII and all other chemicals were obtained from Sigma Chemical Company (St. Louis, MO USA). Bioassay reagents for quantification of total plasma renin and ANP, and a bioassay kit for extraction and quantification of myocardial tissue calcium dependent calcineurin activity were obtained from Sapphire Biosciences (NSW, Australia). Implantable mini-osmotic pumps (Model 2004) were obtained from Alzet (CA, USA).

### Animals and experimental design

Male Sprague Dawley rats (8–9 weeks old, 240–350g) were used for this study. Rats were housed (2–3 animals per cage), at 23°C, 60% humidity on a 14:10 hour light-dark cycle; were fed standard rat chow and allowed access to water *ad libitum*. Animal use and all experimental procedures were approved by James Cook University Animal Ethics Committee according to Australian guidelines for the use and care of laboratory animals (Approval number A2010). Forty-eight rats were randomly divided into six experimental groups (n = 8 per group) and starting body weights were recorded (S1 File). The experimental groups consisted of: Control receiving phosphate buffered saline; Low dose OT (LDOT, 20 ng/Kg/h); High dose OT (HDOT, 100 ng/Kg/h); AngII (40 μg/Kg/h); AngII with LOT and AngII with HDOT. The dose of AngII was based on that used in previous models of hypertension and cardiac hypertrophy induced by AngII infusion [25, 26]. The doses of OT were based on those used in acute studies utilising OT infusion in myocardial infarction [11]. AngII and OT were dissolved in phosphate buffered saline and treatments were administered for 28 days via subcutaneously implanted mini-osmotic pumps. One animal in the AngII only group died suddenly of an unidentified cause before the conclusion of experiments but this did not warrant any changes in the experimental design.

### Osmotic pump implantation

Mini-osmotic pumps were prepared as per manufacturer's instructions. Rats were anaesthetised with isoflurane (5% induction, 2% maintenance, at a flow rate of 2 L/min in oxygen) and an incision approximately 1 cm in diameter was made between the scapulae and pumps were inserted into the incision. Once surgery was completed, wounds were closed using 5.8 mm surgical staples and swabbed with 7.5% w/v iodine to prevent contamination and infection.

### Hemodynamic measurements

Baseline diastolic, systolic and mean arterial blood pressures, and heart rate were measured immediately prior to pump implantation and at day 7 and day 28 after commencement of the experiment. Animals were acclimatized to the restraint apparatus on a heated pad at 37.5°C in a quiet environment under light isoflurane-induced anaesthesia (4% induction, 2% maintenance, at a flow rate of 2 L/min in oxygen). Measurements were taken using a computerised CODA blood pressure monitor (Kent Scientific, CT USA), equipped with an occlusion cuff and a volume pressure recording sensor. Values were recorded until five complete measurements were obtained without the range of the five replicate mean arterial pressures exceeding 15mmHg. All blood pressure measurements were taken between the hours of 6:00 AM-11:00 AM to minimise the impact of diurnal variation. This method has been validated and shown to have good reproducibility and accuracy over the physiological range of blood pressure values in rodents [27].

### Echocardiography

Echocardiograms (M-mode) were obtained 21 days after pump implantation (n = 7–8 per group), under light isoflurane-induced anaesthesia as described above. Rats were positioned right side down on a heated pad and echocardiograms were obtained via the right parasternal short axis view just below the level of the mitral valve using a curved array (3–9 Mhz) probe on a MyLab70 XVision Esaote^®^ ultrasound machine. Left ventricular mass was calculated using the American Society of Echocardiography formula in Eq (1) below and then corrected for variations in body weight where 1.04 is the specific gravity of muscle, LVID_d_ is the left ventricular internal diameter in diastole, IVS_d_ is the interventricular septal diameter in diastole and LVPW_d_ is the left ventricular posterior wall thickness in diastole.

$$1.04\left[{\left({\text{IVS}}_{\text{d}}{+\;\text{LVID}}_{\text{d}}{+\;\text{LVPW}}_{\text{d}}\right)}^{3}-{\left({\text{LVID}}_{\text{d}}\right)}^{3}\right]$$ Eq (1)

Fractional shortening (FS) was calculated according to Eq (2), where LVID_S_ is the left ventricular internal diameter in systole and LVID_d_ is the left ventricular internal diameter in diastole.

$$\left({\text{LVID}}_{\text{d}}-{\text{LVID}}_{\text{s}}\right)\;/\left({\text{LVID}}_{\text{d}}\right)\times 100$$ Eq (2)

For each measured parameter, an average of five cardiac cycles was used by a veterinary echocardiologist who was blinded to the experimental treatment regimens.

### Histological and morphometric analysis of cardiac hypertrophy and renal damage

At day 28, animals were sacrificed by carbon dioxide asphyxiation. Hearts were fixed in diastole by a retrograde apical infusion of 1% potassium chloride, rapidly removed, blot dried, weighed and cut in half along the cross sectional plane. The basal half of the heart and the left kidney were fixed in 10% neutral buffered formalin and embedded in paraffin for histological analysis. Sections of the heart and kidney (5μm) were prepared and stained with hematoxylin and eosin, and Masson’s trichrome, and then photographed under a bright-field with a microscope (Olympus BX43F). Collagen deposition in the posterior wall of the left ventricle and the interventricular septal wall was analysed using Image-J software (NIHealth, MD USA), according to previously described methods [28]. For kidney sections, a histological scoring system based on a semi-quantitative analysis of pathological changes [29] was also used to assess AngII or OT induced changes in a single-blinded manner. Assessed parameters included: glomerular necrosis, tubular degeneration, necrosis and epithelial sloughing, vascular congestion and extravasation, and interstitial fibrosis. The grading system used to assess these pathological changes was based on 0–4 score designed by a pathologist who was blinded to the treatments and was outlined as, 0 = normal with no noticeable histopathological changes; 1 = minor: 0–29%; 2 = moderate: 30–49%; 3 = marked: 50–69% and 4 = severe: 70–100%. Six individual samples were analysed for each of the six experimental groups and histological scores were reported as mean ± standard error of the mean (SEM).

### Quantifying plasma electrolytes, creatinine, urea, ANP and prorenin/renin

At day 28, rats were euthanized by carbon dioxide overdose, blood was collected in heparinised tubes and plasma samples (n = 6 per experimental group) were collected by centrifugation at 2000 x*g*, for 15 min at 4°C and then stored at -20°C prior to analysis. Photometric analysis was performed to determine plasma sodium, potassium, chloride, calcium, magnesium, phosphate, bicarbonate, urea, creatinine, total protein and glutamate dehydrogenase activity using a chemistry analyser (Beckman, AU480). Urea to creatinine ratio was calculated as an indicator for renal failure. Urine flow rate was not measured in this study but an indirect measure of creatinine clearance (mL/min) was used according to a method that utilises plasma creatinine concentration and body mass index in rats [30], using Eq (3).

$$\text{Cr}\left(\text{mL}\;/\;\text{min}\right)={220\times 10}^{\text{-}3}\left(\mu \text{mol}\;/\min\;/\;\text{kg}\right)\times \text{weight}\left(\text{kg}\right)\times {\left[\text{Cr}\left(\mu \text{mol}\;/\text{L}\right)\right]}^{\text{-}1}$$ (3)

Where Cr is plasma creatinine concentration.

Total plasma prorenin/renin concentration was determined using an ELISA assay kit, according to the manufacturer’s instructions (Molecular Innovations) and plasma ANP was quantified by using an ANP ELISA kit [31].

### Calcineurin activity

Frozen heart tissue was thawed and a section of the left ventricle (0.20–0.35g) was isolated and homogenised using a Polytron (Brinkmann PT2100) tissue homogeniser in lysis buffer containing 50 mmol/L Tris (pH 7.5), 0.1 mmol/L EDTA, 0.1 mmol/ L EGTA, 0.2% NP-40, 1 mmol/ L DTT, 100 μmol/ L PMSF, 5 mmol/L ascorbic acid, and protease inhibitor cocktail. The tissue homogenate was centrifuged at 18000 x*g* for 30 min at 4°C and the supernatant was desalted to remove excess phosphate and nucleotides. Calcineurin activity was measured by the rate of dephosphorylation of a synthetic peptide using a calcineurin assay kit according to the manufacturer’s instructions. The released free phosphate was detected using the standard Malachite green colorimetric assay [32]. Relative calcineurin phosphatase activity was expressed as pmol phosphate/μg of protein.

### Statistical Analysis

Normally distributed data were presented as means ± SEM and analysed using one-way analysis of variance with Tukey's post-hoc tests for multiple comparisons. Data that were not consistent with a Gaussian distribution were presented as medians with the interquartile range (IQR) and analysed using the Kruskal-Wallis test paired with Dunn’s post-hoc test for multiple comparisons and the linear mixed effects model to adjust for unequal variance between experimental groups [33]. Changes were considered significant at P < 0.05.

## Results

### Chronic Administration of Oxytocin Accelerated Angiotensin II-induced Hypertension

**Fig 1:**
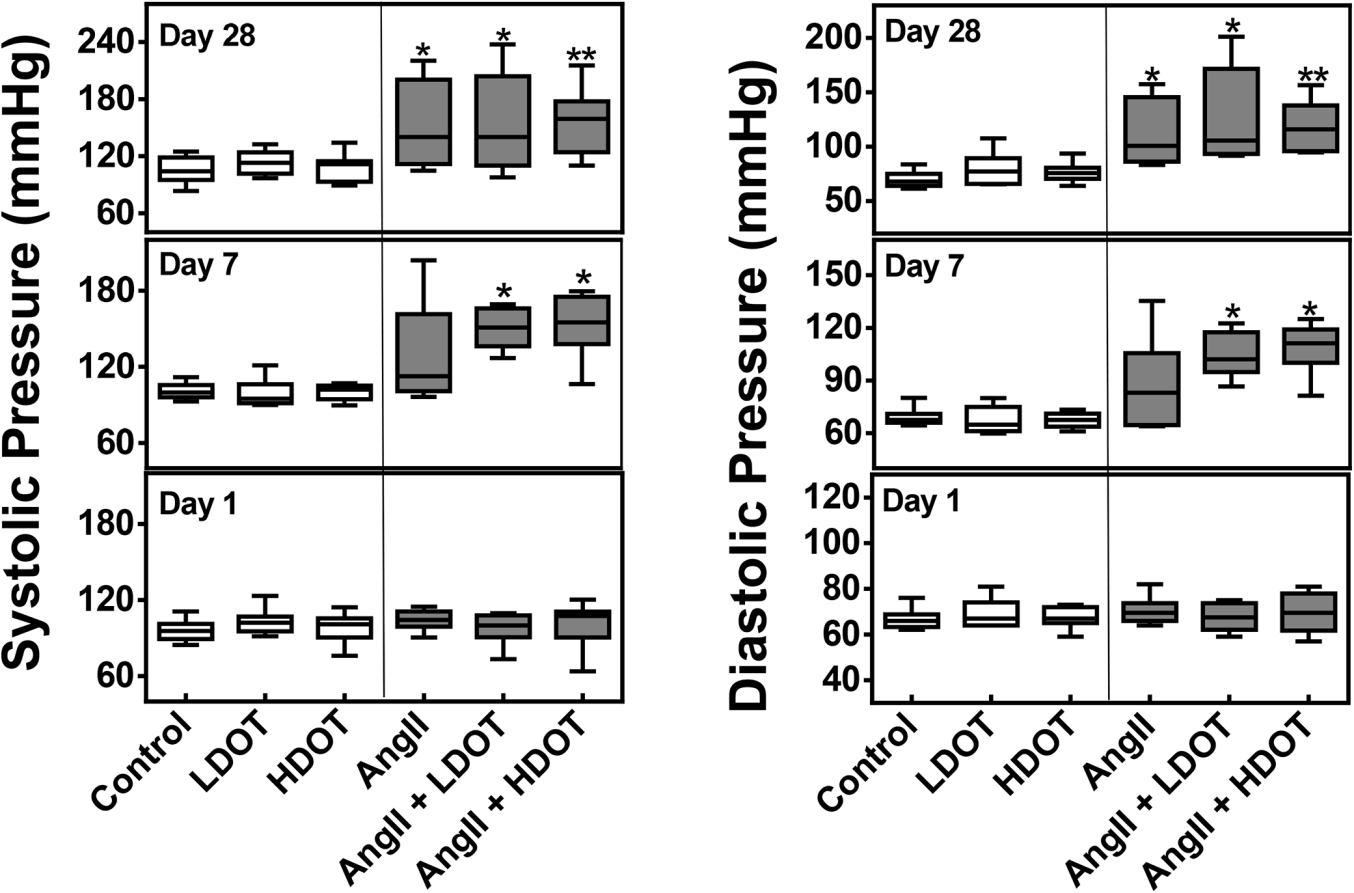
Effects of oxytocin and angiotensin II on systolic and diastolic blood pressure. Systolic and diastolic blood pressure (mmHg) after 1, 7 and 28 days of subcutaneous infusion of saline (control), low dose oxytocin (LDOT), high dose oxytocin (HDOT), angiotensin II (AngII), a combination of angiotensin II and low dose oxytocin (AngII + LDOT) or angiotensin II and high dose oxytocin (AngII + HDOT). Data represent medians with interquartile range (n = 5–8). Asterisks (*) indicate significant difference from the control group on the same day (* P<0.05, ** P<0.01). Clear bars represent saline, low dose oxytocin (LDOT) or high dose oxytocin (HDOT) controls, and filled bars represent angiotensin II, or a combination of angiotensin II and low or high dose oxytocin.

**Fig 2:**
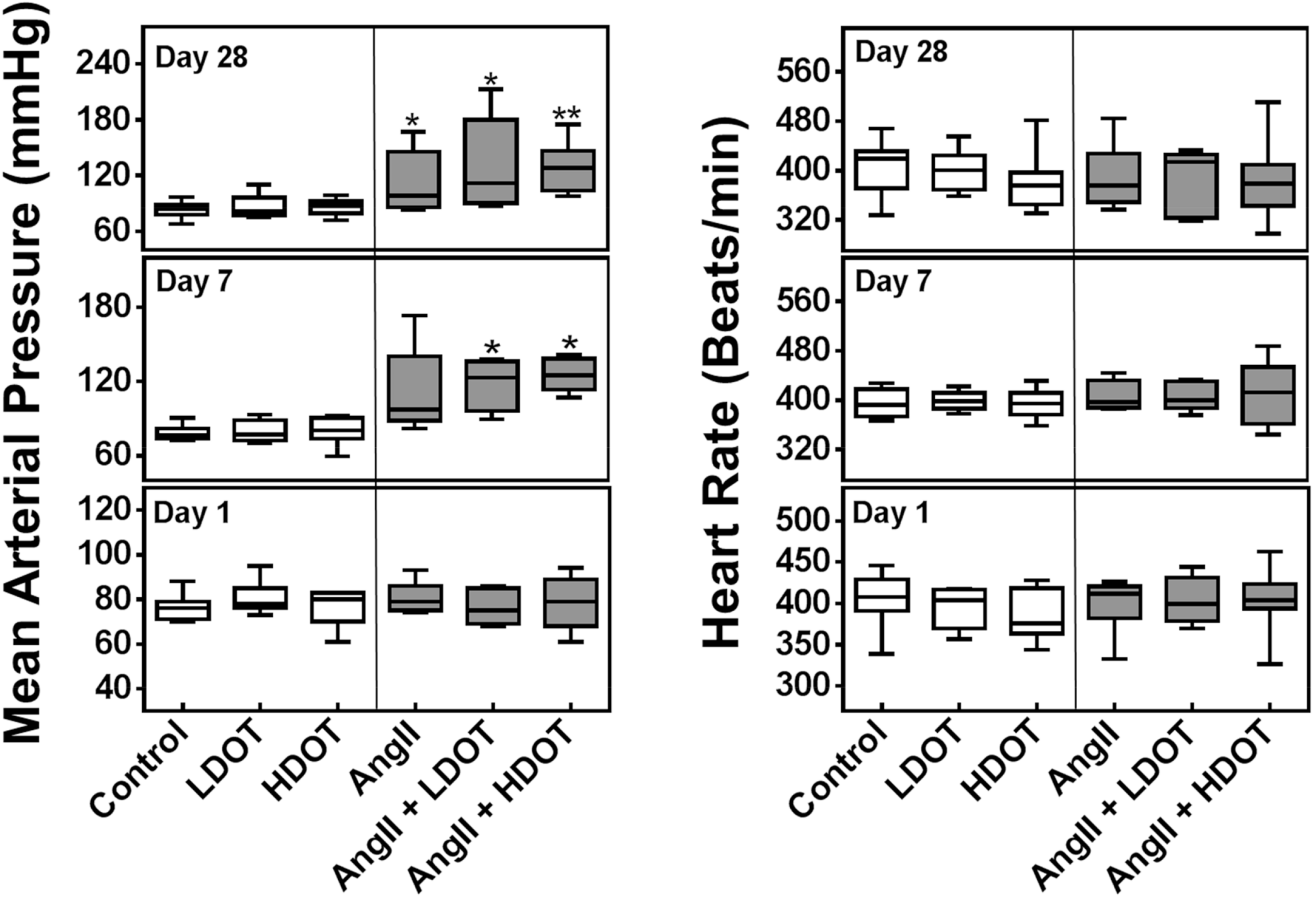
Effects of oxytocin and angiotensin II on mean arterial pressure and heart rate. Mean arterial blood pressure (mmHg) and heart rate (beats/min) after 1, 7 and 28 days of subcutaneous infusion of saline (control), low dose oxytocin (LDOT), high dose oxytocin (HDOT), angiotensin II (AngII), a combination of angiotensin II and low dose oxytocin (AngII + LDOT) or angiotensin II and high dose oxytocin (AngII + HDOT). Data represent medians with interquartile range (n = 5–8). Asterisks (*) indicate significant difference from the control group on the same day (* P<0.05, ** P<0.01). Clear bars represent saline, low dose oxytocin (LDOT) or high dose oxytocin (HDOT) controls, and filled bars represent angiotensin II, or a combination of angiotensin II and low or high dose oxytocin.

Blood pressure and heart rate was measured immediately before pump implantation (day 1) and after 7 and 28 days of OT, AngII or AngII and OT infusion. Administration of only OT at 20 ng/kg/h (LDOT) or 100 ng/kg/h (HDOT) had no effect on any of the measured hemodynamic parameters after 28 days of treatment. At day 7 however, systolic and diastolic blood pressure, and mean arterial pressure was significantly elevated in animals that received both OT and AngII but not AngII only (Figs 1 and 2). Median systolic blood pressure (mmHg) for animals receiving both LDOT and AngII was 151 (146–163) versus control: 100 (98–103); P < 0.05. Diastolic pressure and mean arterial pressure values (mmHg) were 102 (99–112) [LDOT and AngII] versus 68 (67–68) [control; P < 0.05], and 116 (112–131) [LDOT and AngII] versus 76 (75–79) [control; P < 0.05] respectively. Similarly, a significant increase in systolic, diastolic and mean arterial pressure was observed in animals receiving a combination of AngII and HDOT (P < 0.05), but no difference was observed between animals receiving AngII and LDOT, and AngII and HDOT.

At day 28, following the administration of AngII only, AngII and LDOT or AngII and HDOT, a significant increase in systolic, diastolic and mean arterial pressure was observed (Figs 1 and 2). Median arterial pressure values (mmHg) were 85 (79–88) [control] versus 99 (90–129) [AngII only; P < 0.05], 112 (99–152) [AngII and LDOT; P < 0.05] and, 128 (114–140) [AngII and HDOT; P < 0.01]. The hypertensive effects resulting from administration of a combination OT and AngII at day 7 were sustained at day 28 but the administration of AngII together with LDOT or HDOT did not cause a significant change in blood pressure compared to the AngII only group. A significant increase in blood pressure between day 7 and day 28 was only observed in the AngII group (P < 0.05). There were no significant differences in heart rates between any of the treatment groups at day 1, 7 or 28 (Fig 2).

### Effects of Oxytocin on Angiotensin II-Induced Cardiac Hypertrophy and Fibrosis

**Fig 3:**
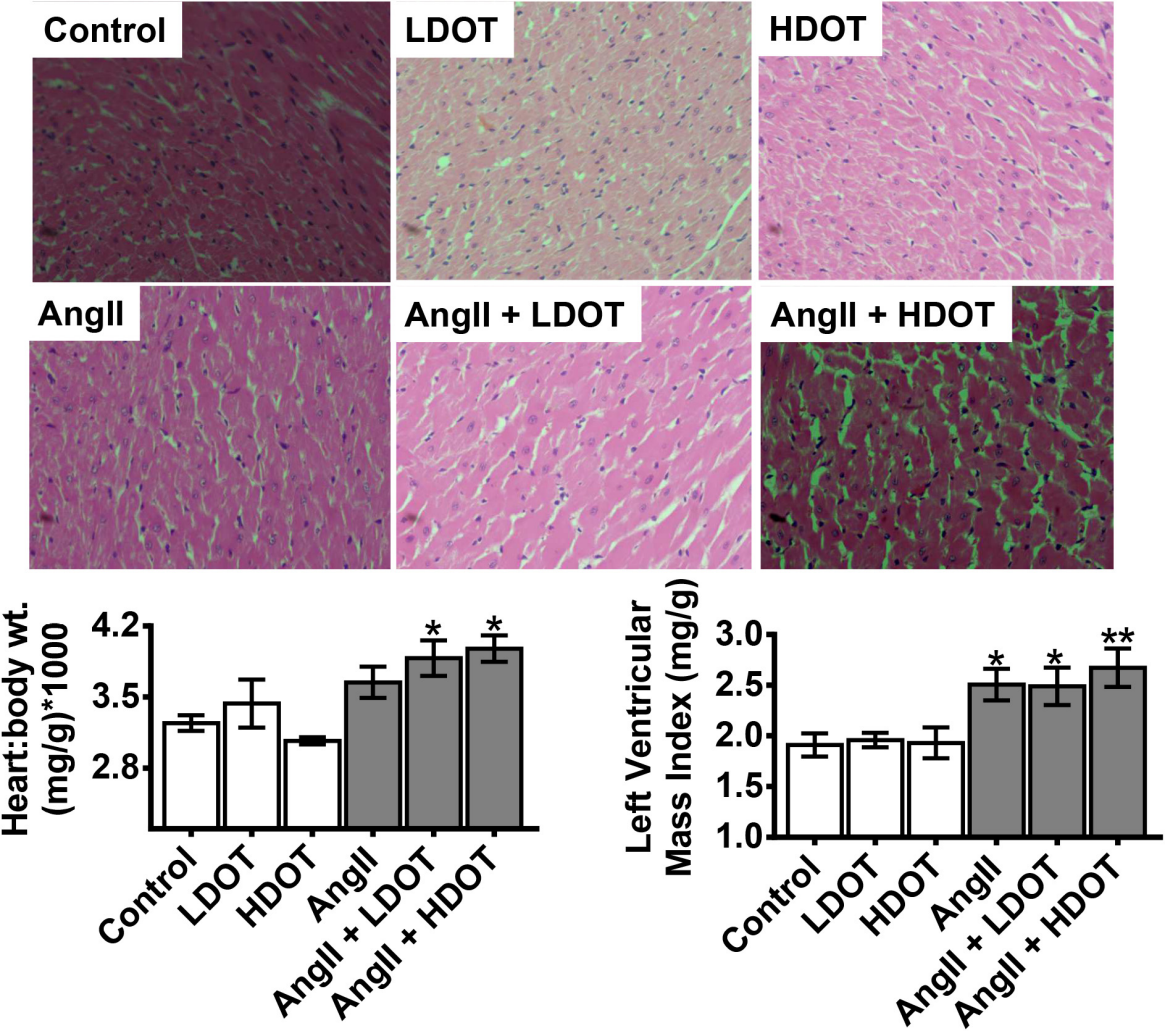
Effects of oxytocin on angiotensin II-induced cardiac hypertrophy. Representative hematoxylin and eosin stained histological sections of the interventricular septal wall of the heart (original magnification x400) showing changes in cardiac myocyte size, left ventricular mass and heart to body weight ratio. Left ventricular mass index and heart weight were quantified at day 21 and 28 respectively following the infusion of saline (control), low dose oxytocin (LDOT), high dose oxytocin (HDOT), angiotensin II (AngII), a combination of angiotensin II and low dose oxytocin (AngII + LDOT) or angiotensin II and high dose oxytocin (AngII + HDOT). Data on bar graphs represent mean ± SEM (n = 6–8). Asterisks (*) indicate significant difference from the control group (* P<0.05, ** P<0.01).

Chronic pressure overload leads to cardiac hypertrophy, cardiomyocyte apoptosis and fibrosis [18]. We assessed effects of OT on AngII induced cardiac hypertrophy and fibrosis. Wet heart weights were measured and normalised to body weight for comparison between treatment groups. Heart weight to body weight ratio was significantly elevated (P < 0.05), only in animals that were administered a combination of AngII and LDOT or AngII and HDOT (Fig 3). Mean heart to body weight ratios (mg/kg) were Control: 3.2 ± 0.2; LDOT only: 3.4 ± 0.7; HDOT only: 3.1 ± 0.1; AngII only: 3.7 ± 0.4; AngII and LDOT: 3.9 ± 0.5; AngII and HDOT: 4.0 ± 0.4. Left ventricular mass index (mg/g) as determined by echocardiography was significantly higher in animals treated with AngII only, AngII and LDOT and, AngII and HDOT compared to the control group. The indices for left ventricular mass (mg/g) were 1.9 ± 0.3 [control] versus 2.5 ± 0.4 [AngII only; P < 0.05], 2.5 ± 0.5 [AngII and LDOT; P < 0.05] and 2.7 ± 0.5 [AngII and HDOT; P < 0.01]. Interventricular septal thickness during diastole and systole was significantly higher only in animals receiving AngII and HDOT compared to control (P < 0.05) but there were no changes in all the other indices of cardiovascular function including percentage FS and ejection fraction in any of the groups (Table 1). Similarly, there were no significant differences in collagen deposition in the posterior wall of the left ventricle and the interventricular septal wall between any of the treatment groups (S2 File).

**Table 1:** Echocardiographic Evaluations of Left Ventricular Dimensions and Function.

	Control	LDOT	HDOT	AngII	AngII + LDOT	AngII + HDOT
**IVS**_**s**_ **(mm)**	2.13 ± 0.10	2.40 ± 0.12	2.18 ± 0.13	2.35 ± 0.20	2.44 ± 0.16	2.76 ± 0.10*
**IVS**_**d**_ **(mm)**	1.36 ± 0.10	1.22 ± 0.07	1.45 ± 0.12	1.57 ± 0.12	1.65 ± 0.13	1.90 ± 0.15*
**LVID**_**s**_ **(mm)**	4.95 ± 0.22	4.96 ± 0.23	5.13 ± 0.12	4.37 ± 0.46	4.33 ± 0.25	4.29 ± 0.30
**LVID**_**d**_ **(mm)**	7.33 ± 0.39	7.49 ± 0.39	7.46 ± 0.16	6.68 ± 0.28	6.79 ± 0.20	6.67 ± 0.29
**PW**_**s**_ **(mm)**	2.70 ± 0.15	3.14 ± 0.13	2.63 ± 0.19	2.83 ± 0.18	2.90 ± 0.14	2.77 ± 0.17
**PW**_**d**_ **(mm)**	2.08 ± 0.18	2.35 ± 0.10	1.90 ± 0.14	2.20 ± 0.22	2.05 ± 0.27	2.00 ± 0.12
**FS (%)**	32.3 ± 1.4	33.0 ± 1.9	31.4 ± 1.6	35.2 ± 4.5	36.3 ± 3.0	35.4 ± 2.4
**EF (%)**	66.1 ± 1.8	68.6 ± 2.9	64.6 ± 2.3	68.7 ± 6.1	70.9 ± 4.3	70.6 ± 3.0

Data represent the mean ± SEM, n = 7–8 per group. Asterisks (*) and bold indicate significant difference compared with control (P < 0.05). LDOT, low dose oxytocin; HDOT, high dose oxytocin; AngII, angiotensin II; IVS_s_, interventricular septum thickness during systole; IVS_d_, interventricular septum thickness during diastole; LVID_s_, left ventricular internal diameter during systole; LVID_d_, left ventricular internal diameter during diastole; PW_s_, left ventricle posterior wall thickness during systole; PW_d_, left ventricle posterior wall thickness during diastole; FS, fractional shortening; EF, ejection fraction.

### Oxytocin Exacerbated Angiotensin II-induced Renal Damage

**Fig 4:**
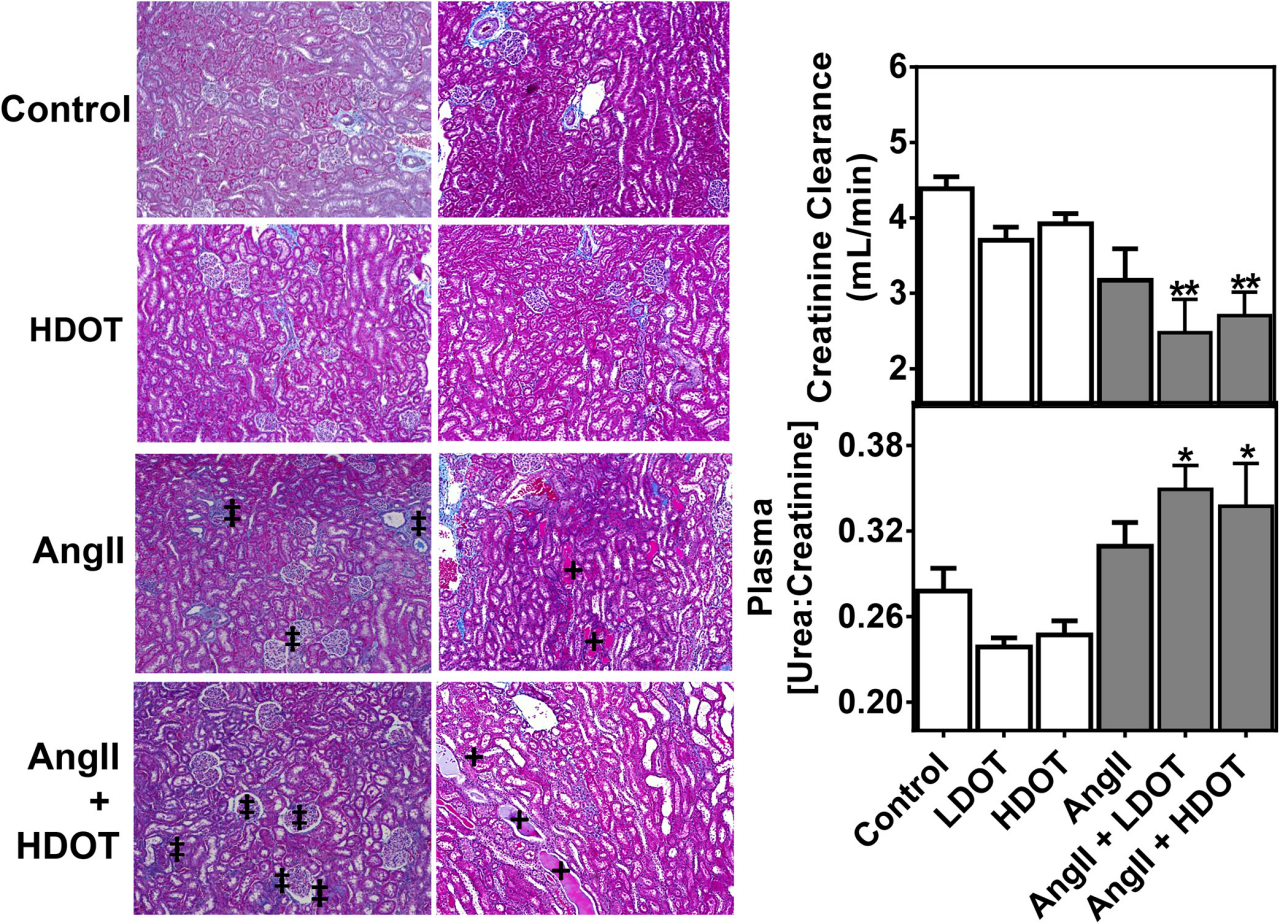
Effects of oxytocin on angiotensin II-induced renal damage. Representative Masson’s trichrome stained histological sections of the renal cortex and medulla showing effects of oxytocin on angiotensin II induced renal damage. Pathological changes in the kidney were assessed by histological evaluation of glomerular necrosis, tubular degeneration, necrosis and epithelial sloughing and interstitial fibrosis (‡), and vascular congestion and extravasation (†). The original magnification was x100. Changes in renal function due to intra-renal damage were also evaluated as plasma urea to creatinine ratio and creatinine clearance. Measurements were made 28 days following the infusion of saline (control), low dose oxytocin (LDOT), high dose oxytocin (HDOT), angiotensin II (AngII), a combination of angiotensin II and low dose oxytocin (AngII + LDOT), or angiotensin II and high dose oxytocin (AngII + HDOT). Data represent the mean ± SEM (n = 6) and asterisks (*) on bar graphs indicate significant difference from the control group (* P<0.05, ** P<0.01).

Kidneys are susceptible to the deleterious effects of chronic hypertension. Effects of OT on AngII-induced hypertension and renal, end-organ damage were assessed by histological evaluations as well as the analysis of plasma urea and creatinine as indicators of renal function. At day 28, plasma urea and creatinine concentrations were significantly elevated only in animals that were treated with both AngII and OT (Table 2 and Fig 4). Mean urea to creatinine plasma concentration ratios were Control: 278 ± 16 versus AngII only: 309 ± 17; AngII and LDOT: 350 ± 16, P < 0.05; AngII and HDOT: 339 ± 27, P < 0.05. The marked increase in plasma urea and creatinine concentration was matched by a corresponding, significant decrease in creatinine clearance in animals that received both AngII and LDOT or Ang II and HDOT (Fig 4). Mean creatinine clearance values (mL/min) were 4.4 ± 0.2 [Control] versus 3.1 ± 0.4 [AngII only], 2.5 ± 0.4 [AngII and LDOT; P < 0.01] and, 2.7 ± 0.3 [AngII and HDOT; P < 0.01]. Significant renal damage (P < 0.05) was also evident on Masson’s trichrome stained histological sections of kidneys from animals that received AngII only, and a combination of AngII and LDOT or AngII and HDOT (Fig 4 and S3 File). Observed pathological effects included tubular epithelial cell necrosis and desquamation, vacuolisation and degeneration of basement membranes, luminal congestion with loss of brush borders, enlargement of Bowman's cavities and, interstitial fibrosis (Fig 4 and S3 File). Histological scores were: 1.2 ± 0.3 in AngII treated animals, 2.1 ± 0.4 in animals that received Ang II and LDOT and 1.7 ± 0.3 in animals that received AngII and HDOT(Fig 4 and S3 File). No noticeable pathological effects were observed in the control, LDOT or HDOT groups.

**Table 2:** Effects of oxytocin and angiotensin II on plasma urea and creatinine concentrations, and plasma electrolytes and osmolality.

	Control	LDOT	HDOT	AngII	AngII + LDOT	AngII + HDOT
**Urea (mM)**	6.6 ± 0.5	6.2 ± 0.3	6.6 ± 0.3	9.2 ± 1.3	13.5 ± 2.3*	15.1 ± 1.9**
**Creatinine (mM)**	0.02 ± 0.001	0.03 ± 0.001	0.03 ± 0.001	0.03 ± 0.001	0.04 ± 0.001*	0.04 ± 0.001*
**Albumin (mg/mL)**	65 ± 1	65 ± 1	67 ± 1	60 ± 1	68 ± 2	64 ± 1
**Na** ^**+**^ **(mM)**	143 ± 1	143 ± 1	144 ± 1	143 ± 1	140 ± 2	142 ± 1
**K** ^**+**^ **(mM)**	7.2 ± 0.6	6.4 ± 0.1	7.2 ± 0.2	5.8 ± 0.4	6.2 ± 0.4	6.6 ± 0.2
**Ca** ^**2+**^ **(mM)**	2.9 ± 0.1	2.9 ± 0.03	3.0 ± 0.1	2.8 ± 0.1	3.0 ± 0.1	2.9 ± 0.1
**Cl** ^**-**^ **(mM)**	97 ± 1	97 ± 1	97 ± 1	96 ± 2	91 ± 2	92 ± 2
**PO**_**4**_^**2-**^ **(mM)**	3.5 ± 0.2	3.1 ± 0.1	3.3 ± 0.1	3.0 ± 0.2	3.2 ± 0.2	3.2 ± 0.3
**HCO**_**3**_^**-**^ **(mM)**	33 ± 2	33 ± 1	34 ± 1	34 ± 2	35 ± 1	36 ± 2
**Osmolality (mM)**	292 ± 2	293 ± 2	294 ± 2	296 ± 1	294 ± 1	294 ± 1

Data represent mean ± SEM, n = 5–6 per group, asterisks (*) and bold indicate significant difference from control group (* P<0.05, ** P<0.01). LDOT, low dose oxytocin; HDOT, high dose oxytocin; AngII, angiotensin-II.

### Effects of Chronic Oxytocin Administration on Plasma Atrial Natriuretic Peptide and Renin Concentration

**Fig 5:**
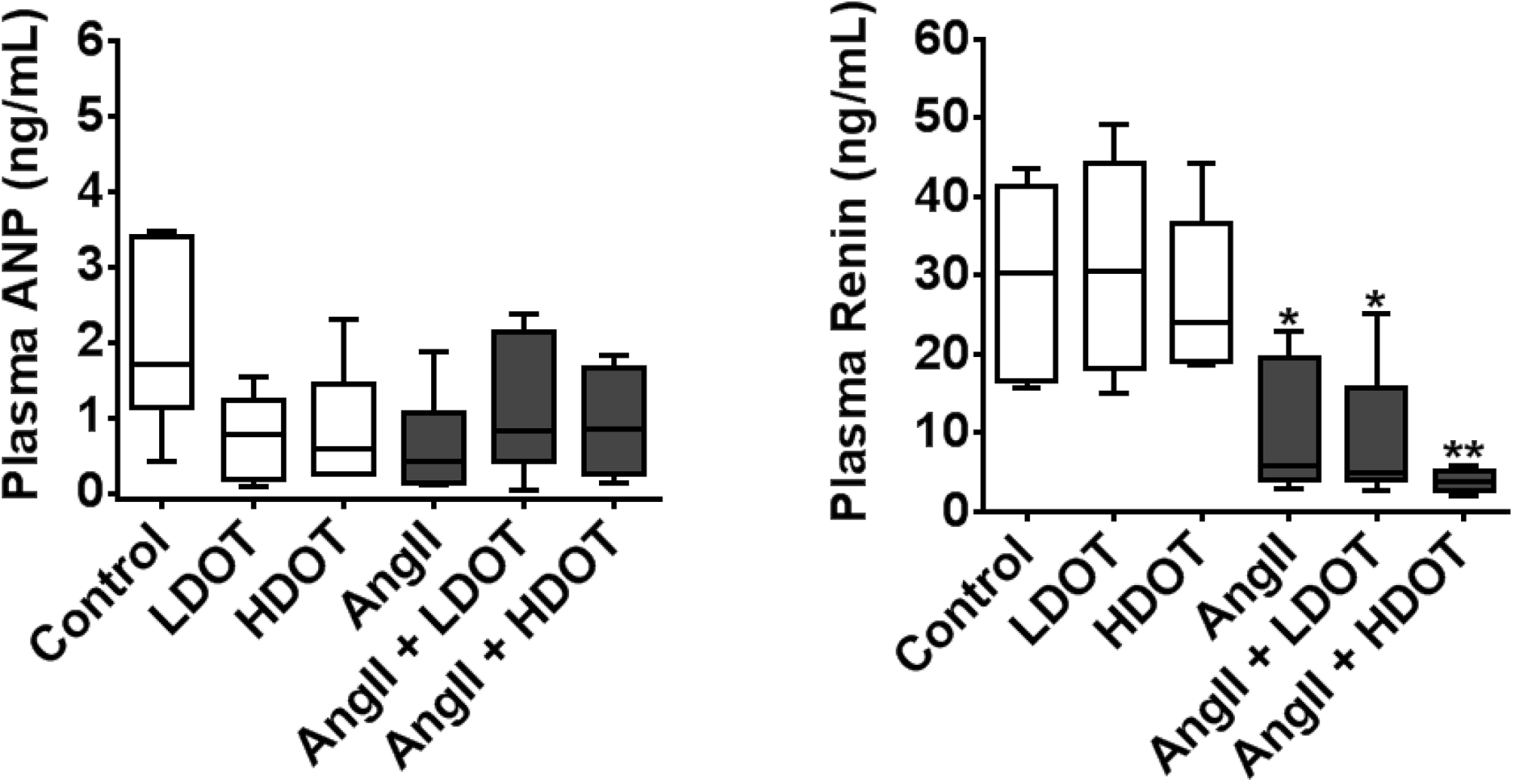
Effects of oxytocin and angiotensin II on plasma ANP and renin concentration. Plasma ANP and, prorenin and renin concentrations were quantified following the administration of saline (control), low dose oxytocin (LDOT), high dose oxytocin (HDOT), angiotensin II (AngII), a combination of angiotensin II and low dose oxytocin (AngII + LDOT) or angiotensin II and high dose oxytocin (AngII + HDOT) for 28 days. Data represent mean ± SEM (n = 5). Asterisks (*) indicate significant difference from the control group (* P<0.05, ** P<0.01).

ANP and renin are important blood pressure regulating factors secreted from the heart and kidneys respectively. In the current study we investigated whether the hypertensive effects resulting from chronic administration of AngII and OT were attributed to sustained changes in circulating levels of ANP and renin. Plasma samples obtained after 28 days of AngII or AngII and OT administration revealed no significant differences in plasma ANP, sodium, potassium and chloride concentrations between any of the treatment groups (Fig 5 and Table 2). In contrast however, total plasma renin and prorenin was significantly decreased (P < 0.05) in all animals that received AngII (Fig 5). Median plasma renin concentrations (ng/mL) were 30 (17–41) [Control] versus 6 (4–16) [AngII only; P < 0.05], 5 (5–11) [AngII and LDOT; P < 0.05] and, 4 (3–5) [AngII and HDOT; P < 0.001].

### Effects of Oxytocin on Angiotensin II-Induced Activation of Phosphatase Calcineurin

**Fig 6:**
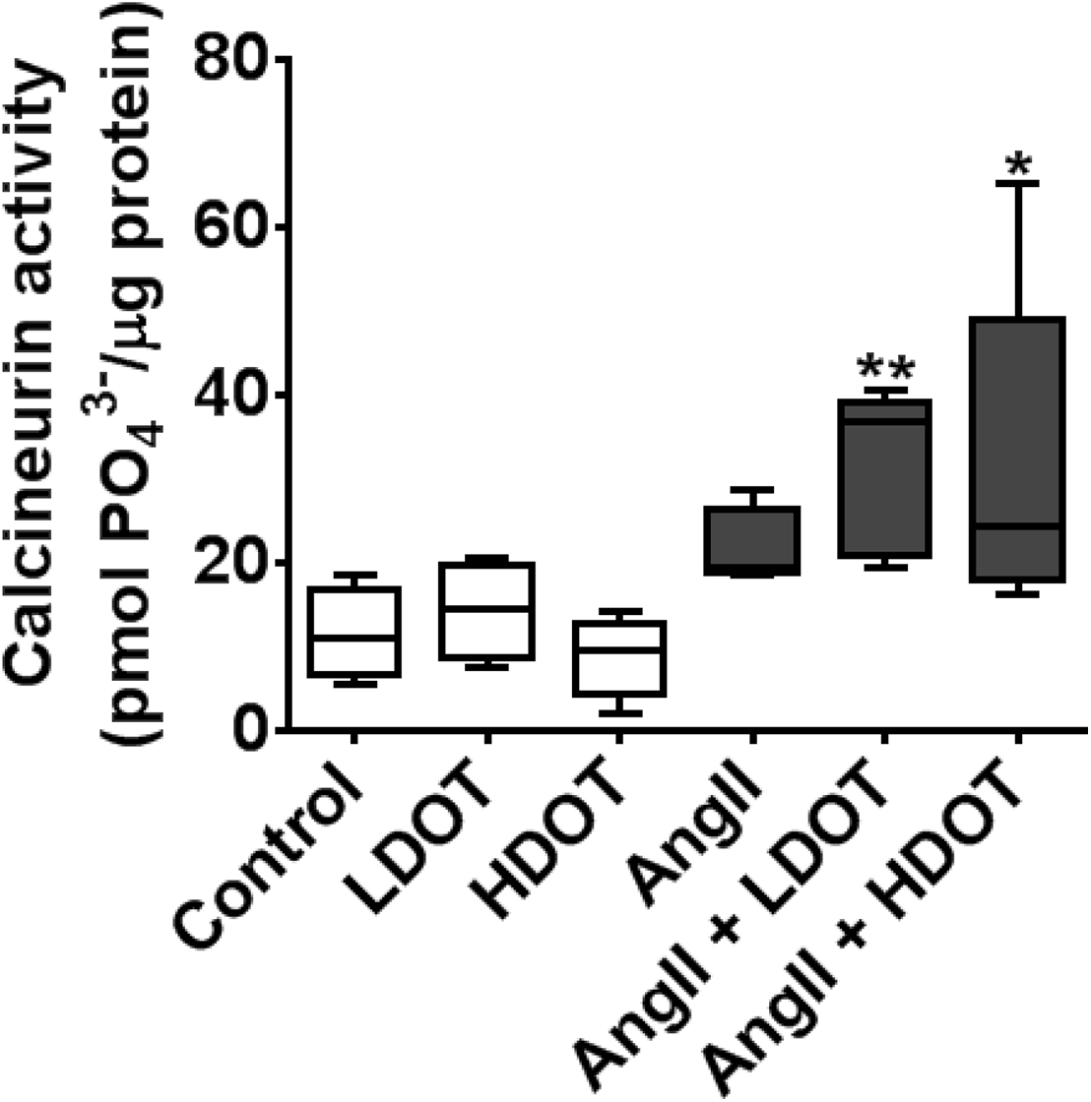
Effects of oxytocin and angiotensin II on myocardial calcineurin activity. Activity of the myocardial calcium-dependent calcineurin phosphatase was quantified following the administration of saline (control), low dose oxytocin (LDOT), high dose oxytocin (HDOT), angiotensin II (AngII), a combination of angiotensin II and low dose oxytocin (AngII + LDOT) or angiotensin II and high dose oxytocin (AngII + HDOT) for 28 days. Data represent mean ± SEM (n = 5). Asterisks (*) indicate significant difference from the control group (* P<0.05, ** P<0.01).

Activation of calcineurin, a calcium-calmodulin-activated serine/threonine phosphatase, plays a critical signal transduction role in the development of pressure overload-induced cardiac hypertrophy, and renal damage. We investigated effects of chronic OT administration on AngII induced activation of calcineurin in heart tissue. The increase in calcineurin phosphatase activity was statistically significant only in animals that received both AngII and, LDOT or HDOT (Fig 6). Median calcineurin phosphatase activities (pmol phosphate/μg protein) were 11 (8–14) [Control] versus 15 (10–18) [LDOT only], 10 (6–11) [HDOT only], 19 (12–22) [AngII only], 36 (22–38) [AngII and LDOT; P < 0.01] and, 25 (19–33) [AngII and HDOT; P < 0.05].

## Discussion

The hypothesis tested in this study was that prolonged systemic administration of OT aggravates the adverse effects of chronic hypertension induced by AngII. Our major observations were that OT accelerated AngII-induced hypertension in a dose dependent manner leading to a compensatory phase of cardiac hypertrophy with increased heart weight and left ventricular mass; but without significant changes in collagen deposition and myocardial fibrosis, or function as determined by evaluation of the left ventricular ejection fraction and fractional shortening. The acceleration of the hypertensive effects of AngII by OT was accompanied by significant kidney damage and compromised renal function. While the prolonged administration of OT did not change plasma concentrations of ANP and renin, or plasma osmolality, it augmented the activation of calcium-dependent protein phosphatase calcineurin, a common signalling mechanism in pressure overload-induced pathological conditions [21, 22].

Chronic hypertension was induced by the subcutaneous infusion of AngII at 40 μg/kg/h for 4 weeks and effects of OT were tested by the simultaneous subcutaneous infusion of OT at 20 ng/kg/h or 100 ng/kg/h for 28 days, to cause chronic oxytocinergic stimulation in male adult Sprague Dawley rats. In previous studies, modelling of cardiovascular disease in Sprague Dawley rats has typically used subcutaneously infused AngII at doses between 10 and 30 μg/kg/h. Following the infusion of 10.5 μg/kg/h of AngII, systolic blood pressure was significantly elevated by day 14 [25], while at 30 μg/kg/h of AngII infusion, systolic blood pressure was significantly elevated by day 7 [34]. The dose and route of AngII administration that we employed in the current study is very comparable to that used by Brink and colleagues [34], however blood pressure was not significantly elevated by day 7 in this study. The male Sprague Dawley rats utilised in our study were younger adults (2 versus 5 months old) and this may have accounted for the delayed development of hypertension. Under these experimental conditions, AngII caused a significant increase in blood pressure by day 28. When the infusion of AngII was combined with OT in the current study, systolic, diastolic and mean arterial blood pressure were significantly elevated at day 7 but by day 28 blood pressure was equally elevated in all animals that received AngII only or AngII and OT. This indicates that hypertension was attained earlier and therefore prolonged, by combining AngII with OT. The sustained hypertension led to a significant, OT dose-dependent increase in heart weight and left ventricular mass in animals that received both AngII and OT. These data are in marked contrast with previous *in vitro* studies where a comparable, low concentration of OT (100 nmoles/L) mitigated AngII-induced hypertrophy and apoptosis in neonatal cardiac myocytes by activating cytoprotective mechanisms including ANP release, endothelial nitric oxide synthase and guanylate cyclase stimulation and increasing intracellular cyclic guanosine monophosphate [24]. This disparity indicates that systemic effects of chronic OT administration may be more complex and thus difficult to predict based on *in vitro* or *ex vivo* studies with acute dosing regimens. Certainly, even with whole animal studies, OT may increase [35, 36] or decrease [36, 37] blood pressure and heart rate depending on dosage and route of administration, length of treatment and, the initial blood pressure status or trigger for hypertension. Here we demonstrate that prolonged subcutaneous administration of OT (20–100 ng/kg/h, 28 days) in itself exerts no changes on heart rate or blood pressure and yet by comparison, intrathecal administration of equivalent doses of OT into the central nervous system caused transient cardiac sympathetic activation and tachycardia, and subsequent lowering of blood pressure via OT receptor mediated stimulation of paraventricular nuclei with spinally projecting neurones [38]. The apparent lack of effect on heart rate in our study may be attributed to the fact that the subcutaneously administered, low dose range of OT had very limited access to the central nervous system mainly because of poor blood-brain barrier permeability and unfavourable pharmacokinetic profiles; this notion is supported by previous studies [39]. It can be argued therefore, that the sub-pressor dose range of OT used in the current study accelerated the hypertensive effects via shared peripheral mechanisms with AngII. At a relatively high plasma concentration, akin to that detected at term pregnancy or during parturition, OT increases blood pressure by activating the vasopressin V1aR receptor [40]. Similarly, AngII is known to stimulate vasopressin release in the circulation and this contributes to increase in blood volume, arterial resistance and elevated blood pressure [41]. It is unlikely however, that involvement of vasopressin and its receptor would serve as the only mechanism to account for the prohypertensive effects of OT in the current study. This is partly because OT has very low intrinsic activity at V1aR vassopressin receptors making it difficult to constitute full functionality at the dose range used here [40]. Opposite hemodynamic challenges associated with hypovolemia and hypotension are accompanied by increased secretion of OT, and the intravenous infusion of OT (125 ng/kg/h) over 120 minutes activates an increase in plasma renin concentration by stimulating OT receptors in macula densa cells of the juxtaglomerular apparatus in the kidney of Sprague Dawley rats [35, 42]. The acute effects of OT on renin release were not re-evaluated in the current study but they do offer a sound rationale for enhancing the renin-AngII system and therefore contributing to the accelerated elevation of blood pressure by OT that we observed. With chronic OT infusion over 28 days however, we observed a significant decrease in total plasma renin concentrations in all animals that received only AngII or AngII and OT. Moreover, in animals that received AngII or AngII and OT treatment, the decrease in total plasma renin was inversely proportional to the elevation in blood pressure at day 7 and 28. This observation is consistent with a form of negative feedback where the increased stimulation of the AngII type 1 receptors reduces renin release from the kidneys as observed to a similar extent in other studies using subcutaneous AngII treatment at a similar dose range [43, 44].

The progression of chronic hypertension to cardiac hypertrophy and congestive heart failure is characterised by an increase in the biosynthesis and release of ANP from the atrial and ventricular myocytes and this serves an important protective mechanism contributing to natriuresis, increased urine volume and lowering of blood pressure [6, 7, 24]. Plasma ANP was therefore measured as a biomarker for cardiac hypertrophy [45, 46], as well as to determine whether OT increased plasma ANP as seen previously [6]. However, when comparable doses of OT (20 or 100 ng/kg/h) were infused subcutaneously for 28 days in the current study, OT, AngII, or a combination of AngII and OT had no effect on total plasma ANP, sodium and potassium concentrations or plasma osmolality. Perhaps this is not surprising because the cardiac hypertrophy that we observed was in a compensatory phase with no signs of overt failure and thus would not be predicted to stimulate significant ANP release. These data also suggest that putative cardiac protective effects of OT in acute conditions such as ischemic reperfusion injury may not be prominent with the chronic administration of OT. We investigated whether chronically administered OT may have exacerbated the effects of AngII via pathological mechanisms mediated by increased intracellular calcium. Indeed, we confirmed that the administration of OT and AngII caused significant activation of calcium-dependent protein phosphatase calcineurin, a canonical early signalling mechanism in pressure overload-induced cardiovascular disease [21, 22]. This indicates that the cardiac myocyte hypertrophy that we observed with AngII only or AngII and OT was an early compensatory phase of a pathological effect but not a normal physiological response.

Kidneys are susceptible to hypertension-induced end-organ damage prompted by reduction of renal perfusion which triggers cellular injury. In this study we show that the acceleration of AngII-induced hypertension by OT corroborated the significant renal damage characterised by tubular epithelial cell necrosis and desquamation, vacuolisation and degeneration of basement membranes, luminal congestion with loss of brush borders, enlargement of Bowman's cavities and, interstitial fibrosis. Thus, these effects may be attributed to the prolonged pressor responses resulting from combining OT and AngII. This is further substantiated by the fact that a significant increase in plasma urea to creatinine ratio and decreased creatinine clearance was observed only in animals treated with both AngII and LDOT or HDOT. As the increase in plasma urea to creatinine ratio and the decrease in creatinine clearance are standard markers for compromised renal function in clinical pathology, we conclude that chronic OT administration exacerbated AngII-induced renal damage. This however, contradicts recent literature which shows that OT administered at a similar dose of 125 ng/kg/h over 12 weeks, exerted cardioprotective effects in leptin receptor deficient (db/db) obese diabetic mice [47]. It was suggested that these changes in db/db mice were partly attributed to decreased body weight gain and adipocyte size, as well as reduced oxidative stress due to the beneficial effects of OT on carbohydrate metabolism [47]. Peripheral OT administration can also reduce appetite in leptin-receptor deficient rats (fa(k)/fa(k)), and inhibit a decrease in energy expenditure due to weight loss [48]. The differences between the results of the current study and that of Plante and co-workers [47], may be due to a number of factors. Firstly, there are prominent species differences between mice and rats and to a great extent, hypertension and renal damage that is seen in experimental models in rats is difficult to induce in mice [49]. Secondly, these studies may collectively suggest that OT is cardioprotective when obesity and diabetes are a major contributing factor, but is not cardioprotective in models of inappropriate RAAS regulation. Thus, it could be hypothesised that OT could be beneficial or detrimental to cardiac health depending on the underlying pathophysiological mechanisms.

### Study Limitations

Based on the experimental design and execution of the current study, useful conclusions can be drawn from the generated data. Nonetheless, there are a few of limitations to this study as outlined below. Firstly, only one model of AngII-induced hypertension was used in rats, and a limited dose range of OT was tested. These observations should be confirmed in other models of chronic hypertension and other animal species with a relatively larger sample size, a wide OT dose range, different routes of OT administration and duration. Secondly, the primary observation of renal damage induced by co-administration of AngII and OT was not confirmed by assessing changes in glomerular filtration rate directly. This was because of the inability to track and quantify total urine output due to the limited access to an adequate number of metabolic cages for rats. Nonetheless creatinine clearance was estimated using a validated method which correlates very well with glomerular filtration rate in Sprague Dawley rats [30]. Our data indicates that the compromised creatinine clearance matched the observed renal damage histologically. Moreover, in animals treated with OT and AngII, the evaluated urea:creatinine ratios (mmol/L:μmol/L), a standard clinical pathology practice indicates a likelihood of intra-renal damage thus supporting these conclusions. Thirdly, we did not quantify changes in plasma AngII and OT. This is partly because subcutaneous infusion of AngII and OT in Sprague Dawley rats has been previously validated [11, 25, 34], and indeed the observed cardiovascular changes indicate that our drug infusions exerted the intended effects. In addition, we only took snapshot readings of blood pressure at day 1, 7 and 28 following OT and AngII infusion. This is a minor limitation as snapshot readings can still provide important information from which meaningful conclusions can be drawn. Our results should be confirmed in future studies utilising telemetry probes to allow for continuous observation of blood pressure. Lastly, detailed mechanisms that underpin some of the conclusions in the current study remain to be determined. The extensive, complex and often overlapping interactions between OT, AngII or vasopressin receptors in maintaining homeostasis in cardiovascular function precluded any meaningful use of receptor antagonist to elucidate mechanisms, but transgenic or OT receptor gene knockout animal models could be utilized to determine whether the observations in the current study were exclusively mediated by the OT receptor. Mechanistically we observed that calcineurin activity was elevated in the myocardium coinciding with the accelerated increase in blood pressure in animals that received both AngII and OT. OT augmented the pathological effects of AngII in the kidneys but kidney specific activation of calcineurin was not demonstrated. While we have access to formalin fixed kidney samples from this study, we realise now that the fixed tissues are not suitable for quantifying renal calcineurin activity and the accompanying downstream biochemical effector pathways. Thus, the current study presents important primary observations relating to prohypertensive effects of OT in an AngII infusion model in rats, but additional studies geared at elucidating detailed molecular mechanisms are warranted.

### Perspectives and conclusions

Intranasal administration of OT is in clinical trials for management of some neuropsychiatric disorders including autism and schizophrenia [2, 3], and a number of studies have indicated that OT may also serve beneficial roles in experimental models of acute myocardial injury [10, 12]. However, the characterisation of cardiovascular effects of OT has been based on acute *in viv*o or *ex vivo* studies and effects of chronic activation of the oxytocinergic system on cardiovascular function are not known. To a great extent, even human clinical trials with OT have been limited to single or sub-acute dosing regimens in normal individuals or patients with behavioural or neuropsychiatric disease [2, 3]. To our knowledge, the current study is the first to investigate effects of prolonged administration of sub-pressor doses of OT on AngII-induced hypertension *in vivo*. We show that OT accelerated AngII-induced hypertension leading to cardiac hypertrophy and, significant damage and compromised function of the kidneys. These effects were associated with the activation of the calcium-dependent calcineurin, a pathological signaling pathway in chronic hypertension. Although we do not show a direct kidney specific effect, this is the first study to show that OT enhances the activation of calcineurin by angiotensin II in cardiovascular tissue. OT may contribute to arterial smooth muscle contraction and hypertension especially in the presence of a pressor agent like angiotensin II in a manner a kin to OT-mediated contraction of uterine smooth muscle. Thus, the acceleration of hypertension by OT was a likely a factor contributing to the increased renal damage. It remains to be determined how our results may relate to the administration of OT via other routes, apply to other species, or models of chronic hypertension but, within the confines and limitations of our experimental design, we conclude that caution should be exercised in the chronic use of OT systemically even at sub-pressor doses in individuals with pre-existing hypertension.

## Supporting Information

S1 File Starting body weights of experimental rats immediately prior to pump implantation. Data represent means ± SEM (n = 8). Ang II, Angiotensin II; LDOT, low dose Oxytocin; HDOT, high dose Oxytocin.
(TIF)

S2 File Masson's trichrome stained histological sections of the posterior wall of the left ventricle. Collagen deposition in the posterior wall of the left ventricle was not significantly different between groups treated with saline (control), low dose oxytocin (LDOT), high dose oxytocin (HDOT), angiotensin II (AngII), a combination of angiotensin II and low dose oxytocin (AngII + LDOT) or angiotensin II and high dose oxytocin (AngII + HDOT) for 28 days. The original magnification was x100.
(TIF)

S3 File Masson’s trichrome stained histological sections of the renal cortex and medulla showing effects of oxytocin on angiotensin II induced renal damage. Pathological changes in the kidney were scored by histological evaluation of glomerular necrosis, tubular degeneration, necrosis and epithelial sloughing and interstitial fibrosis, and vascular congestion and extravasation. The original magnification was x100. Measurements were made 28 days following the infusion of saline (control), low dose oxytocin (LDOT), high dose oxytocin (HDOT), angiotensin II (AngII), a combination of angiotensin II and low dose oxytocin (AngII + LDOT), or angiotensin II and high dose oxytocin (AngII + HDOT).
(TIF)

S4 File The ARRIVE Guidelines Checklist. (PDF)

## Abbreviations

AngII: angiotensin II
ANP: atrial natriuretic peptide
ATP: adenosine triphosphate
HDOT: high dose oxytocin
LDOT: low dose oxytocin
NFAT: nuclear factor of activated T-cells
OT: oxytocin
SEM: standard error of the mean
