# Supplementary figures and images for: Prolonged Subcutaneous Administration of Oxytocin Accelerates Angiotensin II-Induced Hypertension and Renal Damage in Male Rats
Source: PLoS One. 2015 Sep 22;10(9):e0138048. doi: 10.1371/journal.pone.0138048 (PMC4579129; doi:10.1371/journal.pone.0138048)

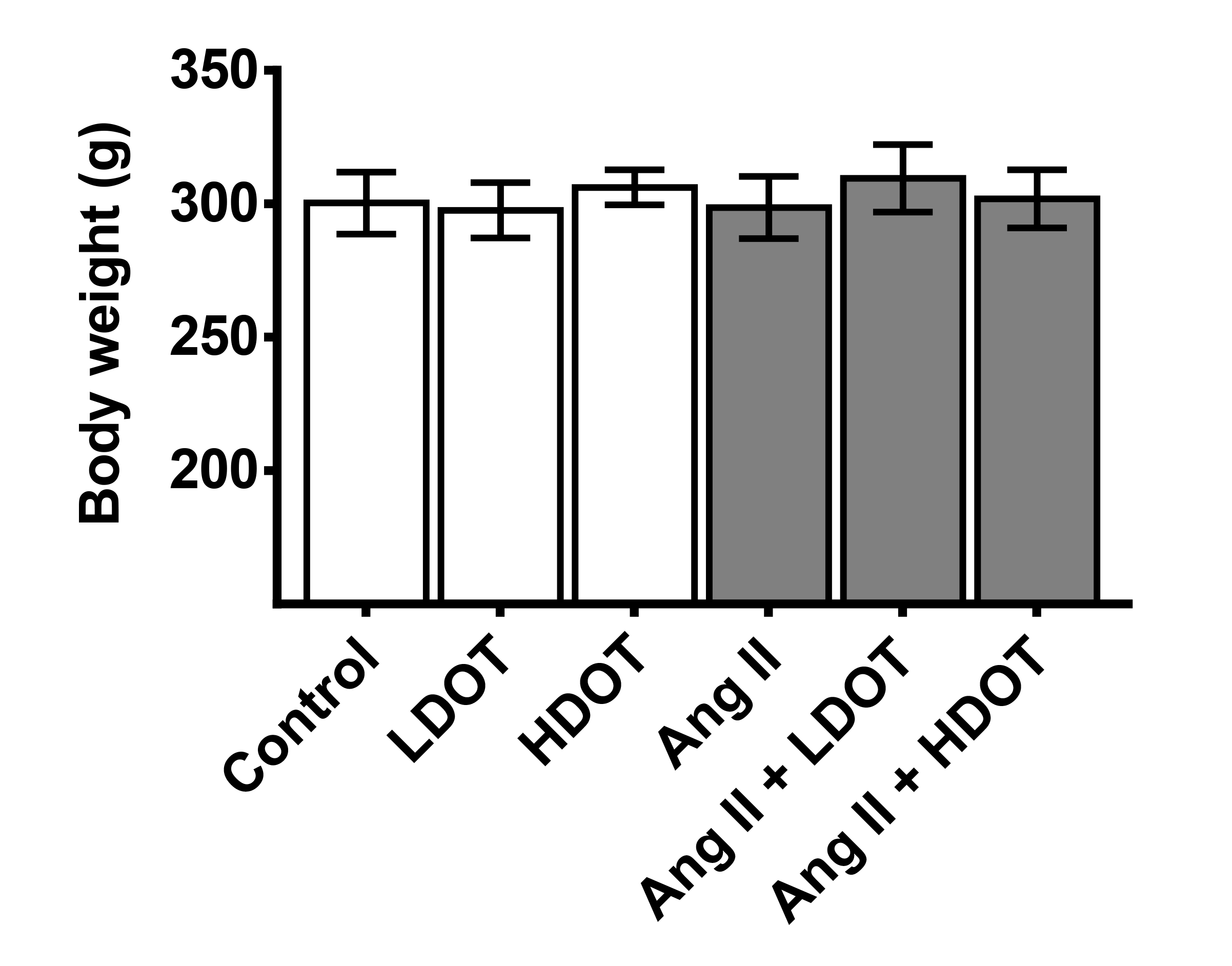

Supplement: S1 File — Data represent means ± SEM (n = 8). Ang II, Angiotensin II; LDOT, low dose Oxytocin; HDOT, high dose Oxytocin. (TIF) [file pone.0138048.s001.tif]

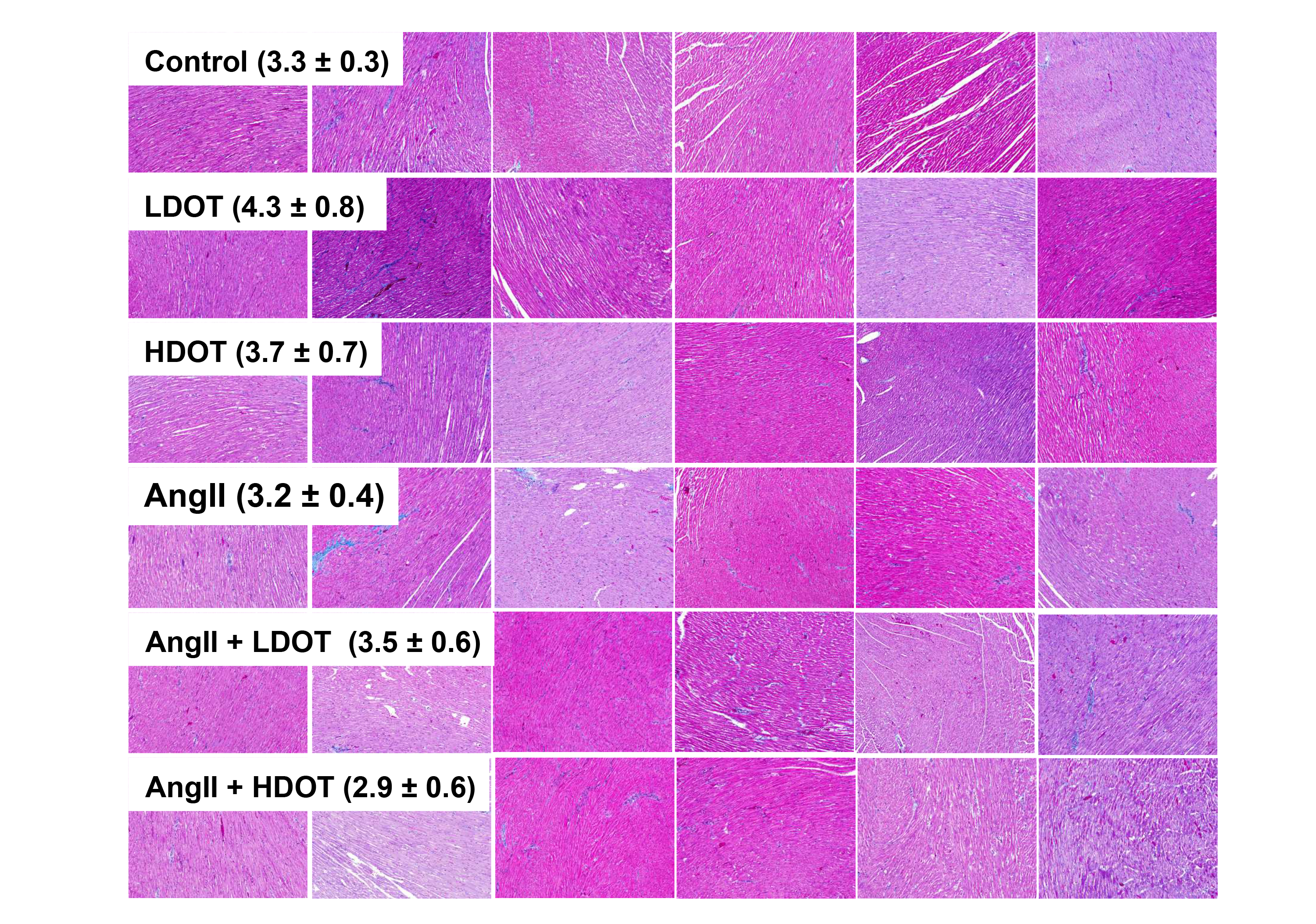

Supplement: S2 File — Collagen deposition in the posterior wall of the left ventricle was not significantly different between groups treated with saline (control), low dose oxytocin (LDOT), high dose oxytocin (HDOT), angiotensin II (AngII), a combination of angiotensin II and low dose oxytocin (AngII + LDOT) or angiotensin II and high dose oxytocin (AngII + HDOT) for 28 days. The original magnification was x100. (TIF) [file pone.0138048.s002.tif]

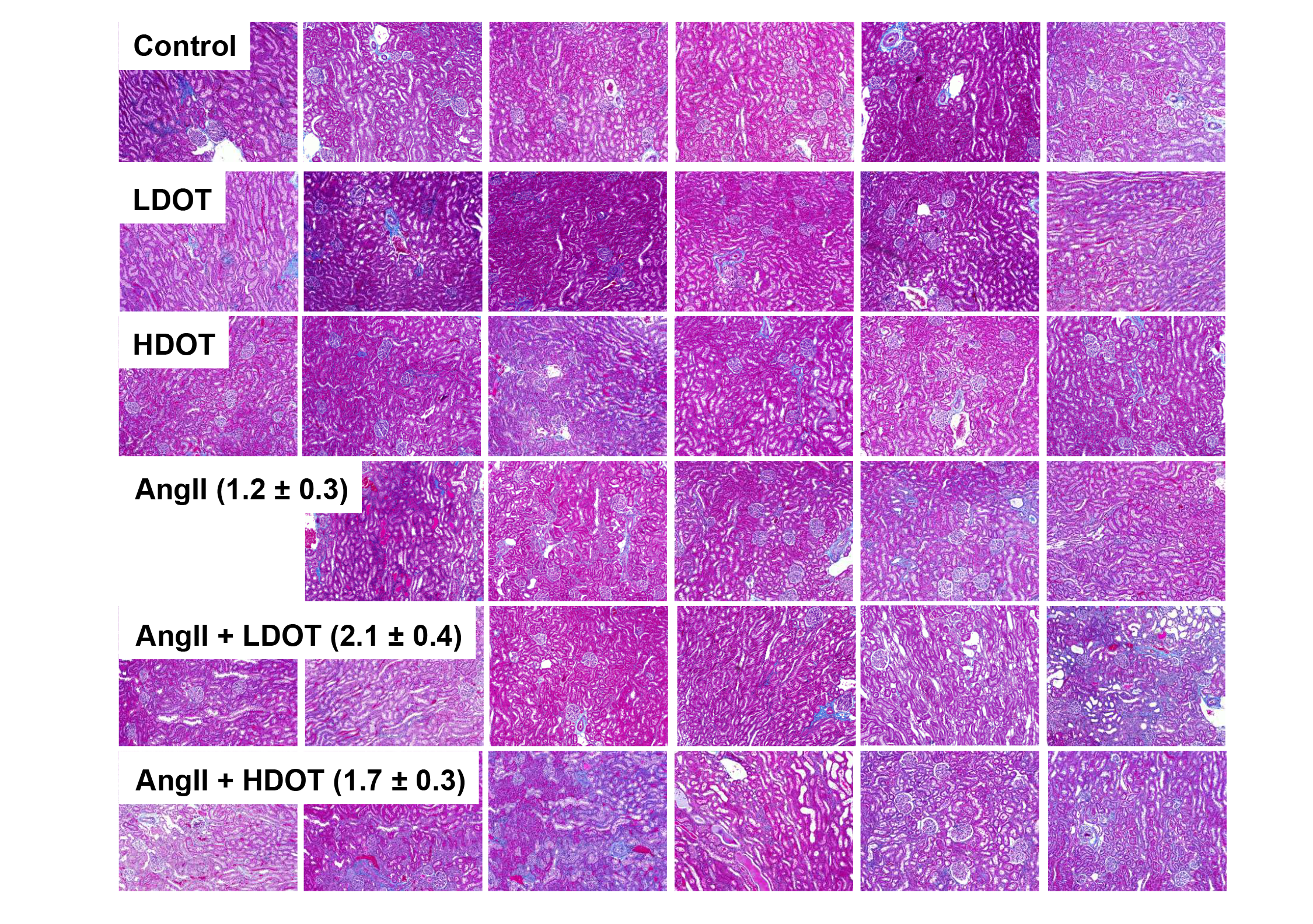

Supplement: S3 File — Pathological changes in the kidney were scored by histological evaluation of glomerular necrosis, tubular degeneration, necrosis and epithelial sloughing and interstitial fibrosis, and vascular congestion and extravasation. The original magnification was x100. Measurements were made 28 days following the infusion of saline (control), low dose oxytocin (LDOT), high dose oxytocin (HDOT), angiotensin II (AngII), a combination of angiotensin II and low dose oxytocin (AngII + LDOT), or angiotensin II and high dose oxytocin (AngII + HDOT). (TIF) [file pone.0138048.s003.tif]
